# First-year all-cause kidney allograft loss: descriptive clinical trajectories of 92 events from a 17-year German transplant center cohort

**DOI:** 10.1186/s12882-026-05306-0

**Published:** 2026-08-24

**Authors:** Ulrich Jehn, Arina Lokschin, Felix Becker, Philipp Houben, Hermann Pavenstädt, Stefan Reuter

**Affiliations:** 1 Nephrology Center Emsland, Lingen, Germany; 2 Department of Medicine D, University Hospital Münster, Münster, Germany; 3 Department of General, Visceral and Transplant Surgery, University Hospital Münster, Münster, Germany; 4 Department of Medicine D Transplant Nephrology, University Hospital Münster, 48149 Münster, Germany

**Keywords:** Kidney transplantation, Graft loss, Death with functioning graft, Death-censored graft loss, Clinical trajectories, Temporal patterns

## Abstract

**Background:**

First-year kidney allograft loss comprises death with a functioning graft (DWFG) and death-censored graft loss (DCGF), each arising from diverse clinical causes. Few studies have examined how endpoint-specific causes are distributed over the first post-transplant year within the same cohort.

**Methods:**

We retrospectively reviewed adult kidney transplant events performed at a German transplant center between 2007 and 2023. For each first-year all-cause graft-loss event, endpoint type and one primary cause were assigned from the documented clinical sequence; additional complications were retained as contributing events. Granular source causes were mapped into higher-order groups defined before examination of their distribution across 0–30, 31–90, and 91–365 day windows.

**Results:**

Among 1400 adult kidney transplant events, 92 first-year all-cause graft-loss events occurred (6.6%, 95% CI 5.4-8.0): 57 DCGF and 35 DWFG. The leading DCGF cause group was vascular/thrombotic or microangiopathic loss (18/57), followed by infection/sepsis (8/57), rejection (7/57), and cardiorenal decompensation/cardiac failure (6/57). Among DWFG events, cardiovascular causes (15/35) and sepsis (9/35) predominated. The proportion of events classified as DWFG increased from 5/32 in the first 30 days to 7/15 at days 31–90 and 23/45 at days 91–365. Six DCGF events were followed by recipient death within the same first year.

**Conclusions:**

First-year all-cause graft loss contained distinct cause and timing patterns. Early losses were predominantly vascular or perioperative DCGF, whereas later events increasingly reflected cardiovascular, infectious, and other medical causes, including DWFG. These findings support phase-specific surveillance and prospective multicenter validation using prespecified assessment of primary and contributing causes.

**Supplementary Information:**

The online version contains supplementary material available at 10.1186/s12882-026-05306-0.

## Background

Kidney transplantation is the preferred kidney replacement therapy for eligible patients with end-stage kidney disease because it offers superior survival and quality-of-life outcomes compared with dialysis [1]. European registry analyses have shown substantial improvements in short-term kidney allograft survival over recent decades; however, early graft loss remains a major adverse outcome for recipients and represents a substantial loss of scarce donor organs [2]. This is particularly relevant in the context of persistent organ shortage, where each graft loss affects not only individual patients but also transplant programs and allocation systems.

The literature on early kidney allograft loss is fragmented by endpoint definition and follow-up interval. Studies restricted to the first postoperative month emphasize vascular thrombosis, primary nonfunction, and technical or surgical complications, whereas first-year studies often examine DWFG, DCGF, or early mortality separately. This makes it difficult to determine whether the underlying causes of these endpoints differ across the first post-transplant year.

Modern cause-of-loss studies and historical analyses have shown that graft loss is often multifactorial and that clinical, histological, immunological, and intercurrent-event information may all contribute to cause assignment [3–8]. For example, Mayrdorfer et al. showed that cardiovascular disease and infection contribute to both DCGF and DWFG, but their analyses did not focus on whether endpoint-specific causes differ in timing within the first year [4, 9].

We therefore sought first to characterize the underlying primary causes of DCGF and DWFG and then to describe how these causes were distributed over clinically interpretable periods within the first post-transplant year. We use the term clinical trajectory descriptively to denote the observed sequence of endpoint type, assigned primary cause, contributing events, and timing. It does not refer to model-derived longitudinal trajectories or prediction modelling.

## Methods

### Study design and setting

This was a retrospective single-center case-series analysis of adult kidney transplant events performed at University Hospital Münster, Germany, between January 1, 2007, and December 31, 2023. All adult transplant events constituted the denominator for first-year all-cause graft loss; events meeting the endpoint were reviewed in detail. The unit of analysis was the transplant event, allowing separate inclusion of retransplantations in the same recipient.

### Data sources and variables

Recipient data were obtained from electronic medical records, including local and external clinical documentation, discharge summaries, operative reports, imaging, microbiology, pathology, and biopsy reports. One-year endpoint status was ascertained for every adult transplant event, including procedures performed in 2023. When local and external records did not provide sufficient information for complete 365-day follow-up, recipients were contacted by telephone. Outcome ascertainment therefore extended for at least 365 days after the last included transplantation; no event was classified as event-free solely because the transplantation period ended on December 31, 2023. Variables comprised demographics, comorbidity, primary renal disease, dialysis vintage, previous transplantation, immunological parameters, immunosuppression, delayed graft function, primary nonfunction, rejection, infection, sepsis, cardiovascular and thrombotic events, bleeding, and other post-transplant complications. Deceased-donor information was obtained from Eurotransplant records; living-donor data were abstracted from local records. Complications occurring before the endpoint were recorded separately from the assigned primary cause.

### Endpoint definitions

The primary endpoint was first-year all-cause kidney allograft loss, defined as permanent return to dialysis, retransplantation, graft nephrectomy, or death with a functioning graft within 365 days after transplantation. DWFG was defined as recipient death while the allograft was functioning and before permanent dialysis, retransplantation, or graft nephrectomy. DCGF was defined as permanent dialysis, retransplantation, or graft nephrectomy in a recipient alive at the time of graft loss. Six recipients who experienced DCGF and subsequently died within the same first year were classified by the first endpoint and additionally recorded as a sequential DCGF-to-death course.

### Primary-cause assignment and harmonization

For each first-year all-cause graft-loss event, the original chart review assigned one primary cause to permit an endpoint-specific cause distribution. The primary cause was the documented event judged to have most directly and proximally resulted in irreversible graft loss or death. Assignment considered contemporaneous clinician diagnoses together with operative findings, imaging, microbiology, histology, and the chronological clinical course. For DWFG, the documented cause of death was assigned and then grouped as cardiovascular, sepsis, intracerebral haemorrhage, gastrointestinal bleeding, malignancy, suicide, or unknown. For DCGF, granular source codes included rejection, vascular thrombosis, thrombotic microangiopathy, bleeding, cardiorenal decompensation/cardiac failure, preservation or surgical complications, urological obstruction, sepsis, viral nephropathy, recurrent disease, and unknown causes.

The source dataset required one primary cause per event, but causal pathways were not assumed to be biologically exclusive. Upstream, concomitant, or downstream complications were retained as contributing events. Thus, in a sequence of BK infection, reduction of immunosuppression, subsequent biopsy-proven rejection, and graft loss, the event supported as the proximate cause of irreversible graft failure was assigned as primary; BK infection and immunosuppression modification remained contributing events. If the documentation and event sequence did not support one dominant proximate cause, the event was classified as unclear/multifactorial. After review of the granular source-code structure, higher-order groups were defined before examination of the timing distribution. Mapping was informed by the available source codes, clinical practice, and established cause-of-loss domains in the literature [3–9]. The original assignments and mappings were reviewed by transplant nephrologists, and discrepancies identified during harmonization were resolved by consensus; no independent blinded adjudication committee or interrater-reliability assessment was performed. Operational rules and source-code mappings are detailed in Supplementary Table S1.

### Timing windows

Time to endpoint was calculated from the recorded decimal year-based variable after conversion to days and grouped a priori into 0–30, 31–90, and 91–365 day windows. These windows represent the perioperative/early technical phase, the early post-discharge and intensified-surveillance phase, and the later first-year follow-up phase, respectively. A value of 0 days denotes an event recorded at transplantation or in the immediate perioperative interval rather than exact zero-hour timing. Three cases coded as 1.0 years were verified as 365-day events and retained in the final window.

### Statistical analysis

The study was designed as a descriptive event-level analysis. Baseline characteristics, clinical events, and causes refer to the 92 first-year all-cause graft-loss events unless explicitly stated otherwise, and percentages use 92 as the denominator. Categorical variables are reported as counts and percentages; continuous variables as mean with standard deviation or median with interquartile range, as appropriate. Wilson score 95% confidence intervals were calculated for selected proportions to quantify precision. Descriptive cumulative-incidence curves for DWFG and DCGF used all 1400 adult transplant events with confirmed 365-day endpoint status; the 1308 events without first-year graft loss were treated as event-free at 365 days. No hypothesis testing, risk-factor modelling, Fine-Gray regression, or multivariable regression was performed. Reporting follows the STROBE statement [10].

### Ethics approval

The study was approved by the Ethics Committee of the Medical Association of Westphalia-Lippe and the Faculty of Medicine of the University of Münster (No. 2014-390-f-N).

## Results

### Cohort and incidence

**Fig. 1:**
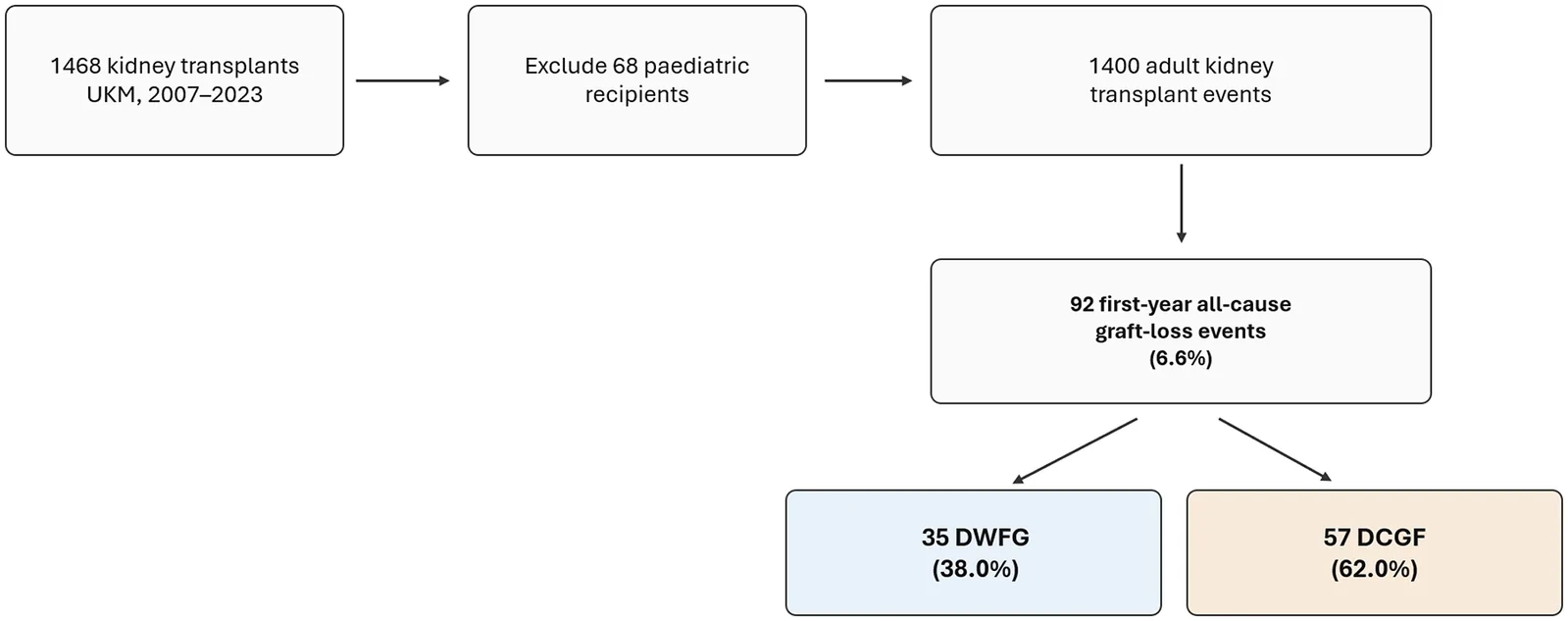
Cohort flow diagram. Adult kidney transplant events at University Hospital Münster from 2007 to 2023. First-year all-cause graft loss was defined as permanent return to dialysis, retransplantation, graft nephrectomy, or death with a functioning graft within 365 days after transplantation

Among 1400 adult kidney transplant events, 92 first-year all-cause graft-loss events occurred (6.6% (95% CI 5.4-8.0)). These events occurred in 90 recipients. Two recipients contributed two separate transplant events after retransplantation during the observation period; these recurrent graft-loss events were distinct from the sequential DCGF-to-death cases described below. Overall, 35 events were DWFG (38.0%) and 57 were DCGF (62.0%). The cohort flow is shown in Fig. 1.

### Baseline, transplant, and selected post-transplant event characteristics

The characteristics of the 92 first-year graft-loss events are summarized in Table 1 to provide clinical context. Detailed recipient, donor, and post-transplant variables were available for the event cohort but not as a comparably structured dataset for transplant events without first-year graft loss. Accordingly, Table 1 is descriptive and is not used for risk-factor inference.

**Table 1:** Baseline, transplant, and selected post-transplant characteristics of the 92 first-year all-cause graft-loss events

Characteristic	Value
Recipient age, years	Median 61.5 (IQR 50.8–67.0); mean 57.8; range 19–78
Male recipient sex	55/92 (59.8%)
Recipient BMI, kg/m^2^	Median 25.9 (IQR 22.0-29.1); mean 26.0
Dialysis vintage, years	Median 5.44 (IQR 2.46-8.00); mean 5.48
Living donor transplantation	12/92 (13.0%)
Deceased donor transplantation	80/92 (87.0%)
ESP transplantation among graft-loss events	25/92 (27.2%)
Donor age, years	Median 62.0 (IQR 48.8–71.3); mean 58.5; range < 1–88
Cold ischaemia time, hours	Median 9.42 (IQR 7.62–12.31); mean 9.99
Warm ischaemia time, minutes	Median 35.0 (IQR 30.0-42.5); mean 37.6
Arterial hypertension	90/92 (97.8%)
Atherosclerotic disease	57/92 (62.0%)
Diabetes mellitus	23/92 (25.0%)
Dyslipoproteinaemia	51/92 (55.4%)
Delayed graft function	43/92 (46.7%)
Primary nonfunction	21/92 (22.8%)
At least one allograft biopsy	78/92 (84.8%)
Biopsy-proven rejection	33/92 (35.9%)
Any documented post-transplant infection	73/92 (79.3%)
Documented sepsis before endpoint	33/92 (35.9%)
Severe cardiovascular event before endpoint	24/92 (26.1%)
Transplant-area thrombotic event	18/92 (19.6%)

Values describe the event cohort only. Primary nonfunction was recorded as an early graft-function phenotype rather than as a mutually exclusive etiologic category; its primary-cause distribution is provided in Supplementary Table S1D. The donor-age range includes one deceased paediatric donor recorded as 0 years (< 1 year)

### Timing and endpoint type

**Fig. 2:**
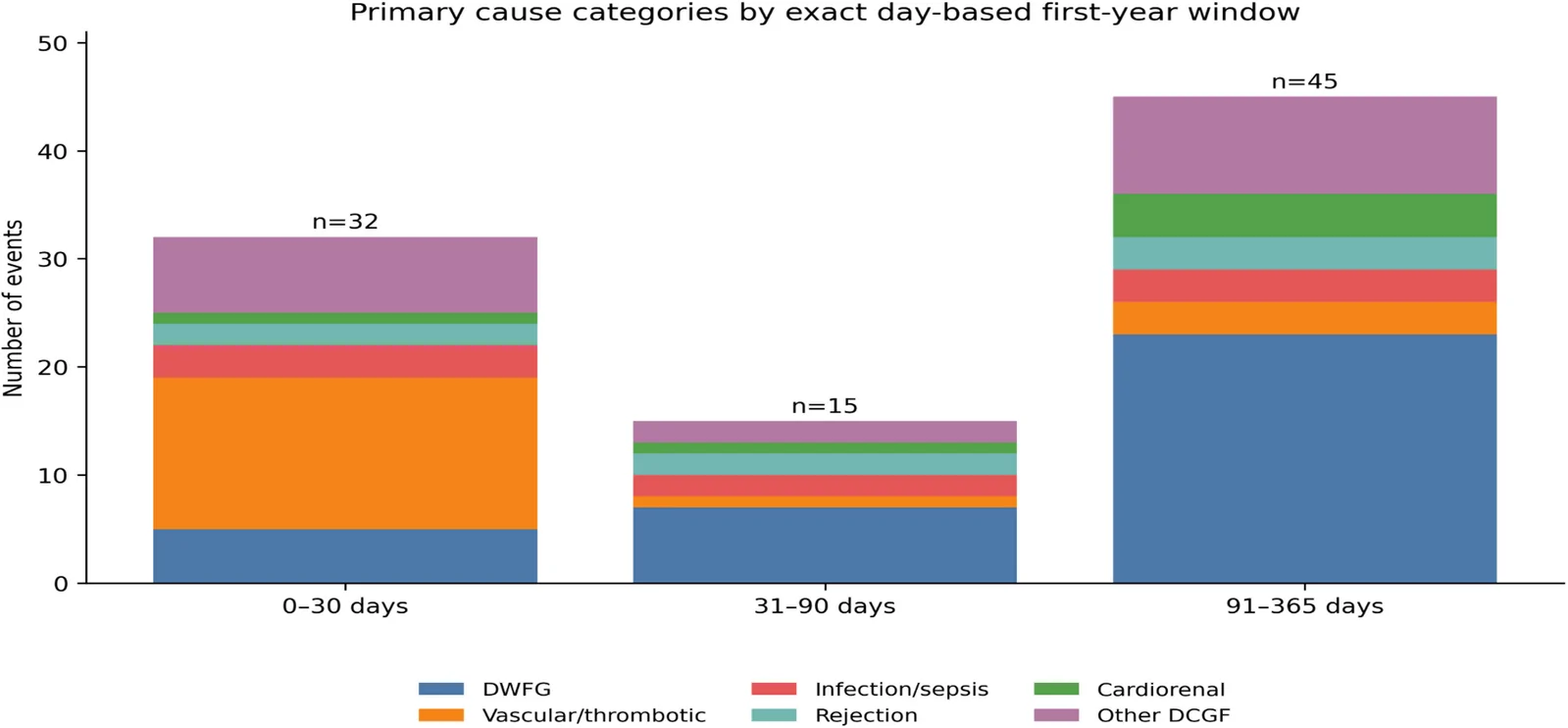
Primary cause categories by the reconstructed first-year clinical time window. The stacked bars show only the primary cause groups. Time windows were derived from the recorded decimal year-based time-to-event variable after conversion to days. Rare death-censored categories were grouped as “Other DCGF” for visual clarity; the full categories are provided in Table 2

**Fig. 3:**
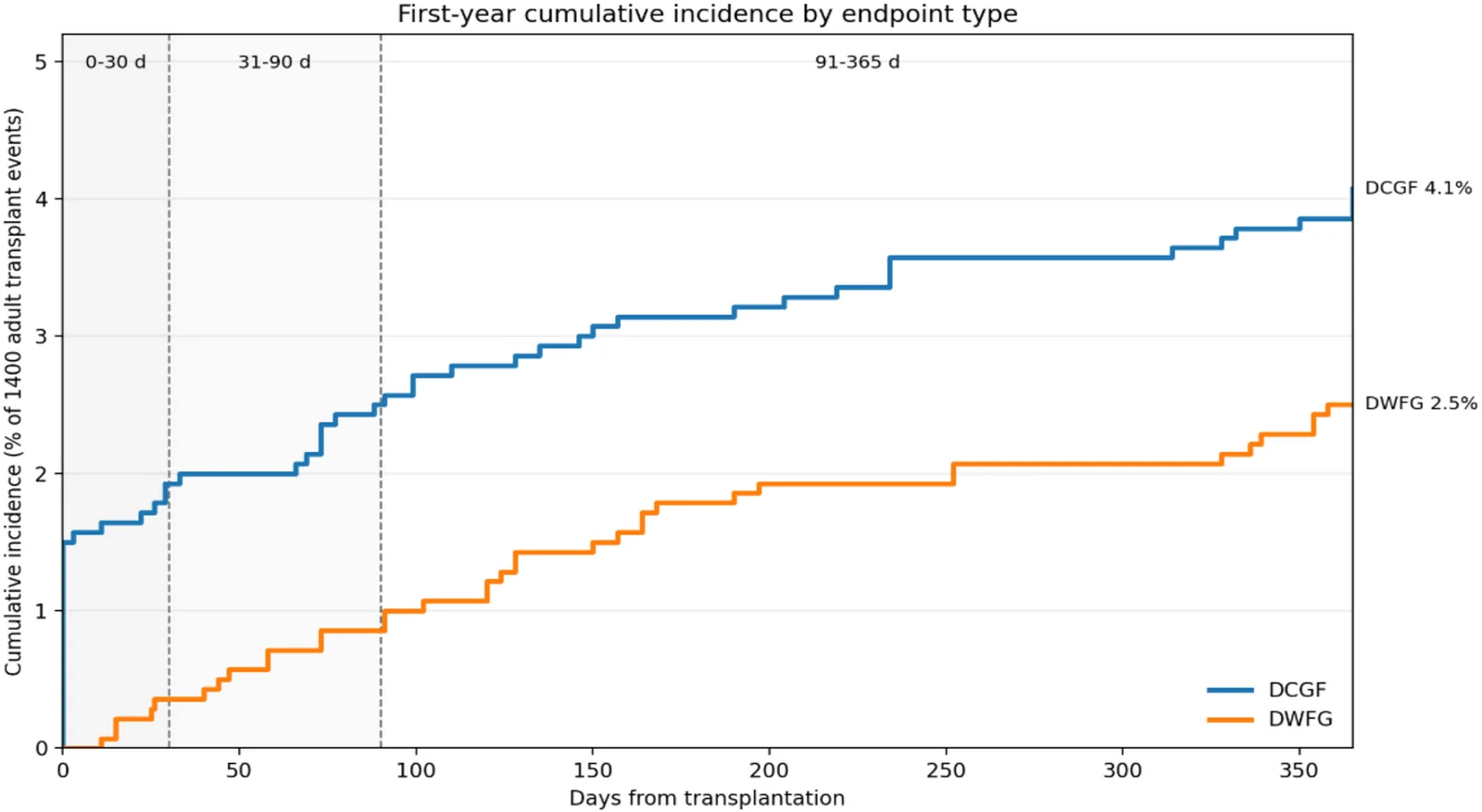
Descriptive cumulative incidence of first-year all-cause graft-loss endpoints by endpoint type. Curves use all 1400 adult kidney transplant events with confirmed 365-day endpoint status; the 1308 transplant events without first-year graft loss were treated as event-free at 365 days. Vertical reference lines indicate the 30-day and 90-day thresholds used for the clinical time-window analysis. DCGF, death-censored graft loss; DWFG, death with functioning graft

Thirty-two events occurred within 0–30 days, 15 within 31–90 days, and 45 within 91–365 days. The proportion classified as DWFG increased from 5/32 events (15.6%) in the first month to 7/15 (46.7%) at days 31–90 and 23/45 (51.1%) at days 91–365; correspondingly, DCGF dominated the first month. Descriptive cumulative-incidence curves showed rapid early accumulation of DCGF, reaching 4.1% at 365 days, whereas DWFG accumulated more gradually and reached 2.5% (Figs. 2 and 3).

### Primary cause categories and cause-specific timing

The 57 DCGF events were assigned to 10 higher-order primary-cause groups, and the 35 DWFG events to seven cause-of-death groups (Tables 2 and 4). Vascular/thrombotic or microangiopathic loss was the leading DCGF group (18/57), followed by infection/sepsis (8/57), rejection (7/57), and cardiorenal decompensation/cardiac failure (6/57). Cardiovascular causes (15/35) and sepsis (9/35) predominated among DWFG events. Six events with no defensible dominant proximate cause remained unclear/multifactorial. Primary nonfunction was treated as an early graft-function phenotype rather than automatically as the primary etiology. Among the 21 events documented with primary nonfunction, the assigned primary cause was vascular/thrombotic or microangiopathic in 12, unclear/multifactorial in 3, rejection in 2, and cardiorenal decompensation, bleeding in the transplant bed, graft malrotation, or sepsis in one each (Supplementary Table S1D). Selected clinical events in Table 1 were more frequent than their corresponding primary-cause assignments because they include contributing events along the clinical pathway.

**Fig. 4:**
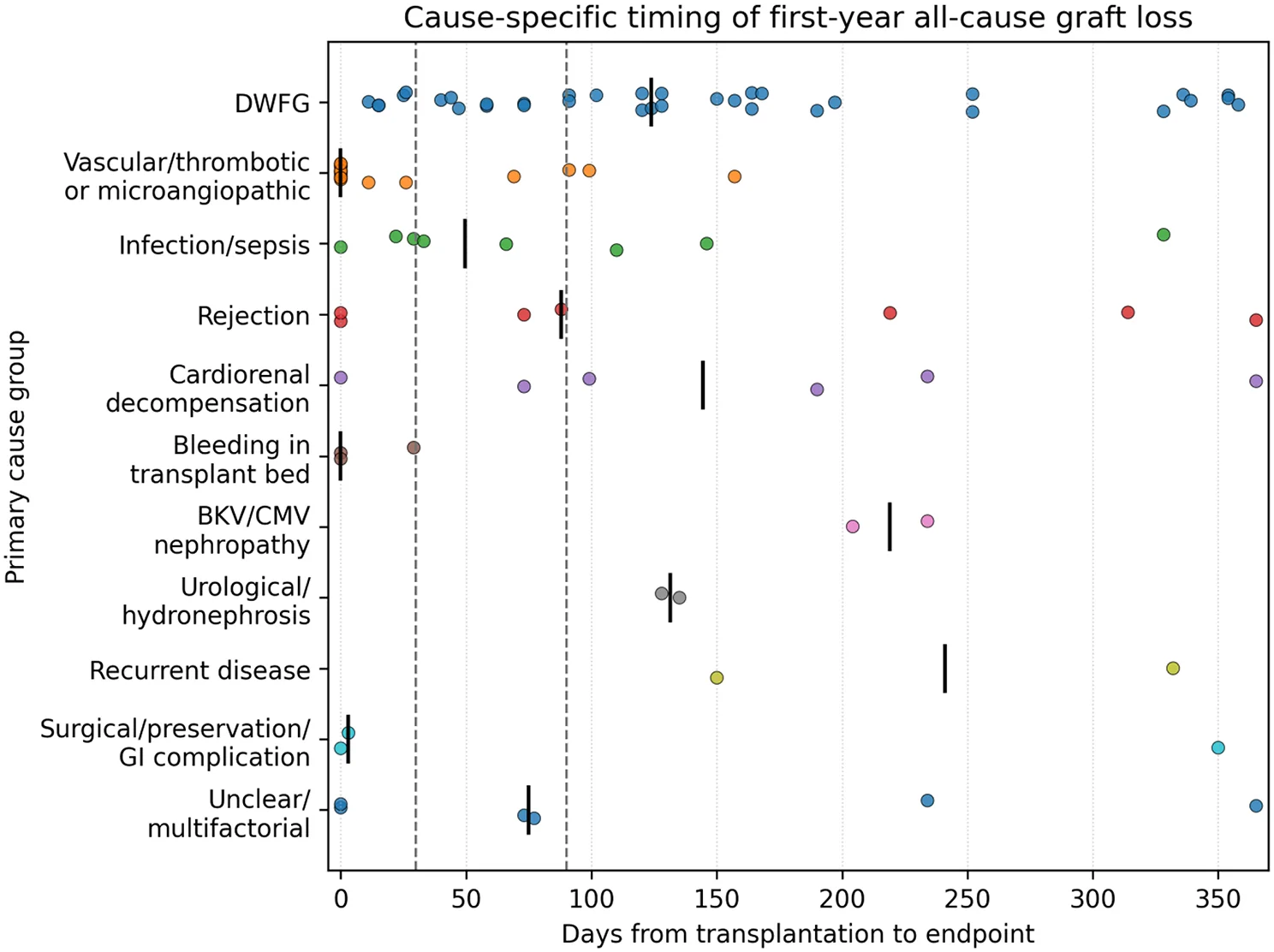
Individual time from transplantation to endpoint by primary cause group. Each dot represents one graft-loss event; vertical dashed reference lines indicate the 1-month and 3-month thresholds used for the clinical time-window analysis. Vertical black markers indicate the group medians. Small groups are shown descriptively and should not be interpreted inferentially

Cause-specific timing differed substantially. Overall median time to endpoint was 83 days (IQR 11–174); median timing was 66 days (IQR 0-150) for DCGF and 124 days (IQR 58–194) for DWFG. Vascular/thrombotic or microangiopathic losses occurred earliest (median 0 days, IQR 0–22), whereas cardiorenal loss, BKV/CMV nephropathy, and recurrent disease clustered later. In a separate subset of six DCGF cases, graft loss was followed by recipient death before the end of the first post-transplant year; five subsequent deaths were attributed to sepsis and one to gastrointestinal bleeding. These cases were classified by the first endpoint and additionally identified as sequential courses (Fig. 4).

For clinical interpretation, the observed combinations of endpoint, primary cause, and timing were summarized into four descriptive patterns (Table 3). These patterns organize the event distribution and are not mechanistic phenotypes or risk classes

**Table 2:** Endpoint and primary cause categories with median time to the endpoint

Endpoint or primary cause category	*n* (% of all 92)	*n* (% of DCGF 57)	Median days to endpoint (IQR)
Death with functioning graft	35 (38.0%)	n/a	124 (58–194)
Vascular/thrombotic or microangiopathic	18 (19.6%)	18 (31.6%)	0 (0–22)
Infection/sepsis	8 (8.7%)	8 (14.0%)	50 (27–119)
Rejection	7 (7.6%)	7 (12.3%)	88 (36–266)
Cardiorenal decompensation / cardiac failure	6 (6.5%)	6 (10.5%)	144 (80–223)
Bleeding in transplant bed	3 (3.3%)	3 (5.3%)	0 (0–15)
BKV/CMV nephropathy	2 (2.2%)	2 (3.5%)	219 (212–227)
Urological/hydronephrosis	2 (2.2%)	2 (3.5%)	132 (130–133)
Recurrent disease	2 (2.2%)	2 (3.5%)	241 (196–287)
Surgical/preservation/GI complication	3 (3.3%)	3 (5.3%)	3 (2-177)
Unclear/multifactorial	6 (6.5%)	6 (10.5%)	75 (18–195)

The first row is the endpoint category death with a functioning graft; its cause-of-death subcategories are shown in Table 4. DCGF, death-censored graft loss; DWFG, death with functioning graft; IQR, interquartile range

**Table 3:** Descriptive clinical patterns derived from endpoint type, timing, and assigned primary cause

Descriptive clinical pattern	Timing / endpoint pattern	Dominant causes or clinical context	Interpretation	*n* in this cohort
Perioperative vascular/microangiopathic pattern	0–30 days; predominantly DCGF	Vascular/thrombotic or microangiopathic loss; bleeding; surgical/preservation complications	Observed concentration of immediate perioperative losses	32
Early mixed clinical pattern	31–90 days; DWFG and DCGF both present	Infection/sepsis, rejection, early graft dysfunction and systemic complications	Mixed endpoint and cause distribution during early follow-up	15
Later first-year medical-event pattern	91–365 days; DWFG increasingly prominent while DCGF persists	Cardiovascular death, infection/sepsis, cardiorenal decompensation, viral nephropathy and recurrent disease	Increasing contribution of recipient-centered and medical events	45
Sequential DCGF-to-death pattern	Variable; DCGF followed by death in the same first year	Severe systemic or infectious trajectories after graft failure	Graft loss followed by death within the same first year	6 (overlap)

The first three rows correspond to the time-window totals. The sequential DCGF-to-death pattern overlaps with the DCGF strata and is not additive

**Fig. 5:**
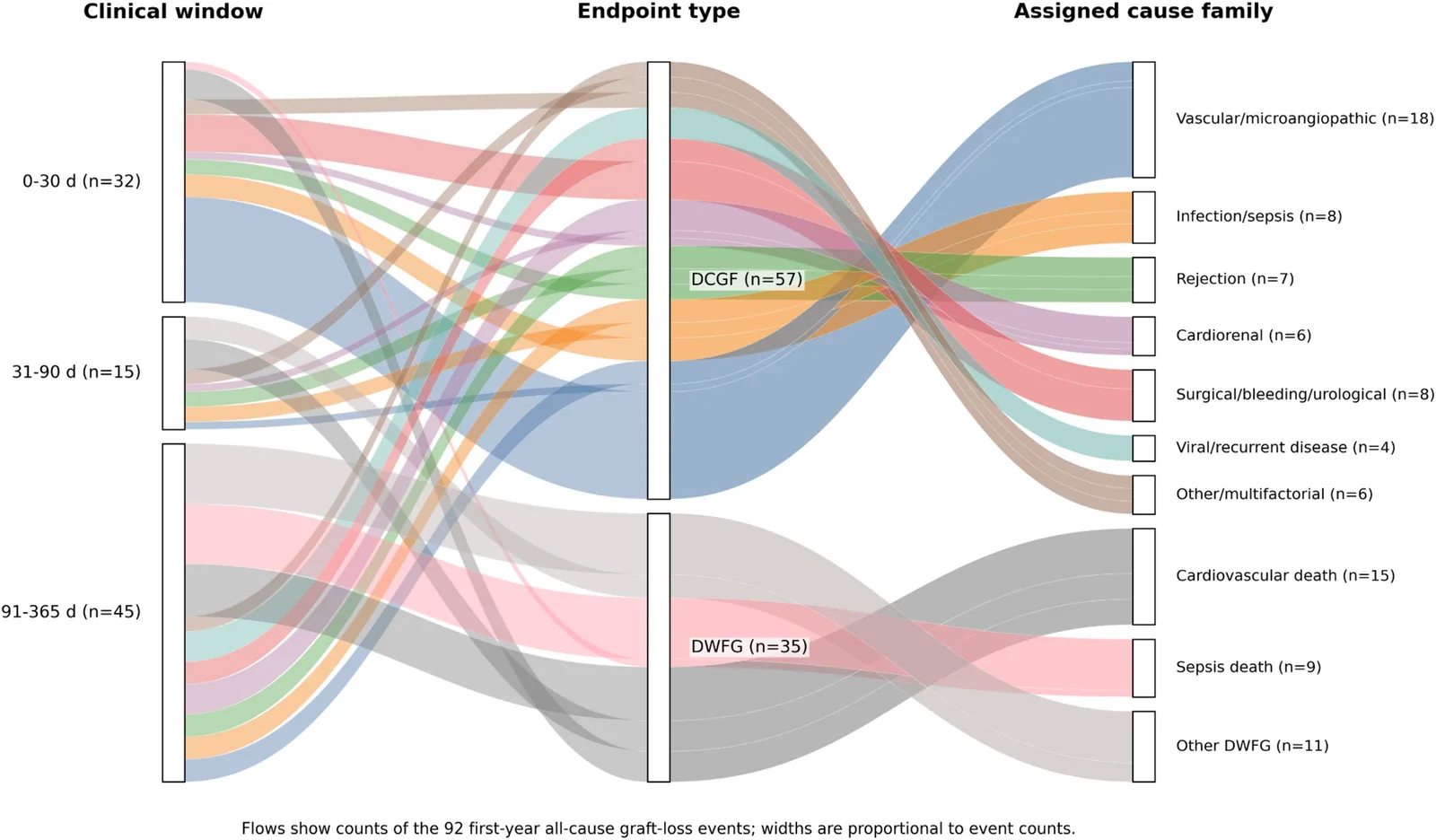
Alluvial visualization of first-year graft-loss event ordering. The plot links clinical time window, endpoint type, and assigned cause family for the 92 first-year all-cause graft-loss events. Flow width is proportional to event count. Cause families combine primary death-censored graft-loss categories and causes of DWFG to provide a compact descriptive overview. DCGF, death-censored graft loss; DWFG, death with functioning graft

A complementary alluvial plot links clinical time window, endpoint type, and assigned cause family for all 92 events (Fig. 5). The plot is a visualization of the observed contingency structure and not an additional statistical model.

### Causes of death with functioning graft

Within DWFG, cardiovascular causes were most frequent (15/35), followed by sepsis (9/35). The remaining causes were intracerebral haemorrhage, gastrointestinal bleeding, malignancy, suicide, and unknown causes (Table 4).

**Table 4:** Causes of death with functioning graft within the first post-transplant year

Cause of DWFG	*n*	% of DWFG
Cardiovascular	15	42.9
Sepsis	9	25.7
Intracerebral haemorrhage	2	5.7
Gastrointestinal bleeding	2	5.7
Malignancy	2	5.7
Suicide	1	2.9
Unknown	4	11.4
Total	35	100.0

## Discussion

### Principal findings

In this study, we sought first to characterize the underlying causes of DCGF and DWFG and then to examine how these causes were distributed within the first post-transplant year. The 57 DCGF events comprised 10 higher-order primary-cause groups, led by vascular/thrombotic or microangiopathic loss, infection/sepsis, rejection, and cardiorenal decompensation. The 35 DWFG events comprised seven cause-of-death groups, led by cardiovascular disease and sepsis.

The second finding was a marked temporal shift. The first 30 days were dominated by DCGF, particularly vascular/thrombotic or microangiopathic loss, whereas after the first month DWFG and other medical causes became increasingly prominent. Six DCGF events were followed by death within the same year, illustrating that graft failure and early mortality may occur sequentially rather than as fully separate outcomes.

### Relation to previous cause-of-loss studies

Detailed cause-of-loss studies by Mayrdorfer, Budde, and colleagues showed that cardiovascular disease, infection, malignancy, and alloimmune injury cross traditional endpoint boundaries and that several contributing causes may coexist within one graft-failure course [4, 9]. Our results are consistent with this overlap. The additional contribution of the present study is the focus on the compressed first year: it describes when endpoint-specific primary causes occurred, rather than treating DCGF and DWFG as uniform outcomes or examining cause domains without their first-year timing.

The distinction between a primary cause and contributing events is therefore important. Each event was counted once in the primary-cause distribution so that cause-specific proportions were interpretable, but this statistical exclusivity does not imply biological exclusivity. Rejection, infection, cardiovascular events, and graft dysfunction could coexist; cases without a defensible dominant proximate cause were retained as unclear/multifactorial rather than assigned artificially.

### Early death-censored graft loss

The strongest temporal signal was the concentration of vascular/thrombotic or microangiopathic DCGF in the first month. This is consistent with studies in which very early graft loss was dominated by allograft thrombosis, primary nonfunction, technical complications, and other perioperative causes [11–13]. The relatively small number of rejection-assigned losses also accords with detailed studies showing that purely alloimmune causes account for only a subset of early death-censored graft failures [8].

These findings support separate reporting of first-month losses and suggest phase-specific quality-improvement targets: immediate assessment of graft perfusion, rapid investigation of absent or deteriorating function, review of technical and anatomical complications, and structured evaluation of thrombotic or microangiopathic injury. Cold ischaemia time is reported only descriptively here; meta-analytic evidence did not identify it as an independent predictor of 1-year graft loss [14].

### Later DWFG and medical causes

After the first month, the event distribution became increasingly recipient- and medical-event centered. Cardiovascular disease and sepsis were the leading causes of DWFG, consistent with previous first-year and longer-term studies of death after kidney transplantation [9, 15–21]. Infection/sepsis also contributed to DCGF, while rejection, viral nephropathy, cardiorenal decompensation, and recurrent disease appeared across the later first year.

This overlap illustrates the competing clinical demands of early transplant care. Reducing immunosuppression during severe infection may increase alloimmune risk, whereas treatment of rejection may increase infectious risk. The sequential DCGF-to-death courses, five of which ended in septic death, further support systematic medical reassessment after early graft failure rather than viewing return to dialysis as the end of the transplant episode. Comprehensive recipient follow-up should integrate graft monitoring with cardiovascular, infectious, and psychosocial care [22]. Cardiorenal decompensation also warrants specific clinical assessment rather than use as a nonspecific label [23].

### Clinical implications and future directions

The study does not establish that individual events were preventable, but it identifies hypotheses for phase-specific prevention. Perioperative strategies should focus on vascular surveillance, perfusion, haemodynamics, and prompt correction of technical complications. After discharge, surveillance should increasingly integrate infection, cardiovascular instability, rejection, and the balance of immunosuppression. Patients returning to dialysis after early graft loss may require intensified infection and cardiovascular follow-up because graft failure can precede death within the same year.

These hypotheses should be evaluated prospectively in a multicenter cohort using denominator-level data, prespecified definitions, independent adjudication, and recording of both primary and contributing causes. Such a design could test whether the observed temporal patterns are reproducible across centers and whether targeted interventions modify graft loss, mortality, or sequential DCGF-to-death courses.

### Strengths and limitations

The study provides granular event-level review of 92 first-year losses, a larger clinically characterized event series than several studies restricted to the first 30 or 90 days, and examines DCGF and DWFG within the same first-year denominator. Its contribution is descriptive rather than epidemiological: the cause and timing information can be read together for each event, but the study does not estimate independent risks.

Detailed baseline and post-transplant variables were available only for the graft-loss events. The study therefore cannot compare event and non-event profiles, identify predictors, or quantify the effect of recipient, donor, or transplant characteristics. The cumulative-incidence curves are descriptive; formal competing-risk regression was not possible because the required structured individual-level data for events without first-year graft loss were unavailable.

Cause assignment was retrospective and depended on the completeness and interpretation of routine clinical documentation. Although the original cause codes and higher-order mappings were clinically reviewed, there was no independent blinded adjudication or interrater-reliability assessment. Multifactorial pathways may therefore be simplified by a single primary-cause distribution despite separate recording of contributing events.

The single-center design introduces selection bias and limits generalizability because local donor, recipient, allocation, perioperative, and follow-up practices shape the observed case mix. In addition, the 2007–2023 period spans changes in clinical practice; a formal era analysis was not possible with the available denominator structure.

## Conclusions

First-year all-cause kidney allograft loss comprised distinct underlying causes whose distribution changed over time. Early events were predominantly vascular, thrombotic, microangiopathic, or perioperative DCGF, whereas later events increasingly reflected cardiovascular, infectious, and other medical causes, including DWFG. These observations support phase-specific surveillance and quality-improvement strategies but do not establish preventability or causal risk factors. Prospective multicenter studies with prespecified assessment of primary and contributing causes are needed to validate the patterns and test whether targeted interventions reduce graft loss, death, and sequential DCGF-to-death courses.

## Supplementary Information

Below is the link to the electronic supplementary material.

Supplementary Material 1: Supplementary Table S1. Operational definitions and source-code mapping used for endpoint and primary-cause assignment

## Data Availability

The data underlying this study contain potentially identifiable clinical information and are not publicly available. De-identified aggregate data may be made available from the corresponding author upon reasonable request and subject to institutional and ethical restrictions.

## Declarations

### Use of generative artificial intelligence

The authors declare that ChatGPT (OpenAI) was used to assist with language editing, structural consistency checks, and manuscript formatting. All analyses, scientific interpretation, and final manuscript content were reviewed and approved by the authors.

## Abbreviations

BKV: BK polyomavirus
CI: Confidence interval
CMV: Cytomegalovirus
DCGF: Death-censored graft loss
DWFG: Death with a functioning graft
ESP: Eurotransplant Senior Program
IQR: Interquartile range
STROBE: Strengthening the Reporting of Observational Studies in Epidemiology

## References

[1] Tonelli M, Wiebe N, Knoll G, Bello A, Browne S, Jadhav D, et al. Systematic review: kidney transplantation compared with dialysis in clinically relevant outcomes. Am J Transpl. 2011;11:2093–109. 10.1111/j.1600-6143.2011.03686.x 21883901

[2] Coemans M, Süsal C, Döhler B, Anglicheau D, Giral M, Bestard O, et al. Analyses of the short- and long-term graft survival after kidney transplantation in Europe between 1986 and 2015. Kidney Int. 2018;94:964–73. 30049474 10.1016/j.kint.2018.05.018

[3] Sellarés J, de Freitas DG, Mengel M, Reeve J, Einecke G, Sis B, et al. Understanding the causes of kidney transplant failure: the dominant role of antibody-mediated rejection and nonadherence. Am J Transpl. 2012;12:388–99. 10.1111/j.1600-6143.2011.03840.x 22081892

[4] Mayrdorfer M, Liefeldt L, Wu K, Rudolph B, Zhang Q, Friedersdorff F, et al. Exploring the complexity of death-censored kidney allograft failure. J Am Soc Nephrol. 2021;32:1513–26. 33883251 10.1681/ASN.2020081215 PMC8259637

[5] Redondo-Pachón D, Calatayud E, Buxeda A, Pérez-Sáez MJ, Arias-Cabrales C, Gimeno J, et al. Evolution of kidney allograft loss causes over 40 years (1979–2019). Nefrologia (Engl Ed). 2023;43:316–27. 37507293 10.1016/j.nefroe.2023.07.003

[6] Mulley WR, Hughes PD, Collins MG, Pilmore HL, Clayton PA, Wyld ML, et al. Defining causes of death-censored kidney allograft failure: a 5-year multicentre ANZDATA and clinical cross-sectional study. Nephrol (Carlton). 2024;29:930–40. 10.1111/nep.14397 PMC11579561 39349052

[7] El-Zoghby ZM, Stegall MD, Lager DJ, Kremers WK, Amer H, Gloor JM, et al. Identifying specific causes of kidney allograft loss. Am J Transpl. 2009;9:527–35. 10.1111/j.1600-6143.2008.02519.x 19191769

[8] Van Loon E, Senev A, Lerut E, Coemans M, Callemeyn J, Van Keer JM, et al. Assessing the complex causes of kidney allograft loss. Transplantation. 2020;104:2557–66. 32091487 10.1097/TP.0000000000003192

[9] Mayrdorfer M, Liefeldt L, Osmanodja B, Naik MG, Schmidt D, Duettmann W, et al. A single centre in-depth analysis of death with a functioning kidney graft and reasons for overall graft failure. Nephrol Dial Transpl. 2023;38:1857–66. 10.1093/ndt/gfac327 PMC10387383 36477607

[10] von Elm E, Altman DG, Egger M, Pocock SJ, Gøtzsche PC, Vandenbroucke JP, et al. The Strengthening the Reporting of Observational Studies in Epidemiology (STROBE) statement: guidelines for reporting observational studies. Ann Intern Med. 2007;147:573–7. 17938396 10.7326/0003-4819-147-8-200710160-00010

[11] Phelan PJ, O’Kelly P, Tarazi M, Tarazi N, Salehmohamed MR, Little DM, et al. Renal allograft loss in the first post-operative month: causes and consequences. Clin Transpl. 2012;26:544–9. 10.1111/j.1399-0012.2011.01581.x 23050275

[12] Hamed MO, Chen Y, Pasea L, Watson CJ, Torpey N, Bradley JA, et al. Early graft loss after kidney transplantation: risk factors and consequences. Am J Transpl. 2015;15:1632–43. 10.1111/ajt.13162 25707303

[13] Helanterä I, Räihä J, Finne P, Lempinen M. Early failure of kidney transplants in the current era-a national cohort study. Transpl Int. 2018;31:880–6. 29341290 10.1111/tri.13115

[14] Foroutan F, Friesen EL, Clark KE, Motaghi S, Zyla R, Lee Y, et al. Risk factors for 1-year graft loss after kidney transplantation: systematic review and meta-analysis. Clin J Am Soc Nephrol. 2019;14:1642–50. 31540931 10.2215/CJN.05560519 PMC6832056

[15] Farrugia D, Cheshire J, Begaj I, Khosla S, Ray D, Sharif A. Death within the first year after kidney transplantation-an observational cohort study. Transpl Int. 2014;27:262–70. 24138318 10.1111/tri.12218

[16] Yu MY, Kim YC, Lee JP, Lee H, Kim YS. Death with graft function after kidney transplantation: a single-center experience. Clin Exp Nephrol. 2018;22:710–8. 29159528 10.1007/s10157-017-1503-9 PMC5956041

[17] Awan AA, Niu J, Pan JS, Erickson KF, Mandayam S, Winkelmayer WC, et al. Trends in the causes of death among kidney transplant recipients in the United States (1996–2014). Am J Nephrol. 2018;48:472–81. 30472701 10.1159/000495081 PMC6347016

[18] Ying T, Shi B, Kelly PJ, Pilmore H, Clayton PA, Chadban SJ. Death after kidney transplantation: an analysis by era and time post-transplant. J Am Soc Nephrol. 2020;31:2887–99. 32908001 10.1681/ASN.2020050566 PMC7790214

[19] Helve S, Helanterä I, Laine M, Nieminen T, Finne P, Helve J. Trends and specific causes of cardiovascular mortality after kidney transplantation in Finland. Clin J Am Soc Nephrol. 2024;19:355–63. 37962909 10.2215/CJN.0000000000000360 PMC10937022

[20] Vinson AJ, Singh S, Chadban S, Cherney D, Gaber O, Gill JS, et al. Premature death in kidney transplant recipients: the time for trials is now. J Am Soc Nephrol. 2022;33:665–73. 35292438 10.1681/ASN.2021111517 PMC8970447

[21] Merzkani MA, Bentall AJ, Smith BH, Benavides Lopez X, D’Costa MR, Park WD, et al. Death with function and graft failure after kidney transplantation: risk factors at baseline suggest new approaches to management. Transpl Direct. 2022;8:e1273. 10.1097/TXD.0000000000001273 PMC8759617 35047660

[22] Kidney Disease. Improving Global Outcomes (KDIGO) Transplant Work Group. KDIGO clinical practice guideline for the care of kidney transplant recipients. Am J Transpl. 2009;9(Suppl 3):S1–155. 10.1111/j.1600-6143.2009.02834.x 19845597

[23] Waiser J, Knebel F, Rudolph B, Wu K, Müller E, Sanad W, et al. Renal allograft loss caused by cardiorenal syndrome: frequency and diagnosis. Transplantation. 2015;99:1208–15. 25539469 10.1097/TP.0000000000000501

