# Supplementary material for: First-year all-cause kidney allograft loss: descriptive clinical trajectories of 92 events from a 17-year German transplant center cohort
Source: BMC Nephrol. 2026 Aug 24;27:499. doi: 10.1186/s12882-026-05306-0 (PMC13505005; doi:10.1186/s12882-026-05306-0)
Supplement: Supplementary file 1 [file 12882_2026_5306_MOESM1_ESM.docx]

**Supplement**

Purpose. This supplement specifies how the retrospective source review distinguished endpoint type, primary cause, contributing events, higher-order cause groups, timing, and primary nonfunction. The source dataset required one primary cause per event for descriptive counting; this does not imply that the underlying clinical pathway was biologically or clinically monocausal.

# Table S1A. Operational sequence and rules

| **Step** | **Operational definition** | **Evidence and decision rule** | **Handling of overlap or uncertainty** |
| --- | --- | --- | --- |
| 1. Event eligibility | A kidney transplant event was eligible if permanent dialysis, retransplantation, graft nephrectomy, or death with a functioning graft occurred within 365 days. | Transplant and follow-up records were reviewed. Every adult transplant event had ascertainable 365-day endpoint status; when records were insufficient, recipients were contacted by telephone. The transplant event, rather than the recipient, was the unit of analysis. | Separate retransplantations in the same recipient could contribute separate events. The administrative transplantation period ended in 2023, but outcome ascertainment continued for at least 365 days after the last included transplantation. |
| 2. Endpoint type | DWFG was death before permanent dialysis, retransplantation, or graft nephrectomy while the graft remained functioning. DCGF was permanent dialysis, retransplantation, or graft nephrectomy in a recipient alive at graft loss. | The first irreversible endpoint determined classification. | A DCGF event followed by death within the same year remained classified as DCGF and was additionally recorded as a sequential DCGF-to-death course. |
| 3. Primary cause | One event was selected as the primary cause for descriptive cause-specific counting. | The primary cause was the documented event judged to have most directly and proximally resulted in irreversible graft loss or death. | The single primary-cause field was a counting convention and did not imply biological exclusivity. |
| 4. Information considered | Cause assignment integrated the documented clinical sequence. | Contemporaneous clinician diagnoses, discharge and death documentation, operative findings, imaging, microbiology, histology/biopsy findings, and timing were considered. | No rigid evidence hierarchy was imposed when the record clearly documented the endpoint-producing event. |
| 5. Contributing events | Complications occurring before or around the endpoint were recorded separately from the primary cause. | Examples include infection, rejection, cardiovascular events, bleeding, delayed graft function, primary nonfunction, and immunosuppression changes. Primary nonfunction was treated as an early graft-function phenotype and not automatically as an etiologic primary cause. | Contributing events could coexist across several domains and were not forced into the primary-cause count. |
| 6. Multifactorial sequence | A dominant proximate cause was assigned only when supported by documentation and event ordering. | Example: after BK infection, immunosuppression reduction, biopsy-proven rejection, and graft loss, the event supported as the proximate cause of irreversible loss was primary; BK infection and treatment modification remained contributing events. | If neither BK nephropathy nor rejection, or any other candidate cause, could be prioritized, the event was classified as unclear/multifactorial. |
| 7. Higher-order harmonization | Higher-order harmonization before timing analysis | After review of the granular source-code structure, higher-order groups were defined before examination of the timing distribution. Mapping was informed by the source codes, clinical practice, and established cause-of-loss domains in the cited literature. | The groups were not prospectively specified before data collection. Mapping reduced sparse categories but did not alter the original event sequence. |
| 8. Timing | Events were assigned to 0-30, 31-90, or 91-365 days after conversion of the recorded decimal-year variable to days. | The windows were specified a priori as perioperative/early technical, early post-discharge, and later first-year phases. | Three cases coded as 1.0 years were verified as 365-day events. |
| 9. Clinical review | Original cause assignments and mappings were reviewed by transplant nephrologists; discrepancies identified during harmonization were resolved by consensus. | Review focused on consistency between source codes, clinical documentation, and higher-order mapping. | No independent blinded adjudication committee or formal interrater-reliability assessment was available. |

# Table S1B. Mapping of granular DCGF source codes to higher-order groups

| **Granular source cause** | **Higher-order group used in the manuscript** | **Clarification** |
| --- | --- | --- |
| Rejection | Rejection | Assigned when rejection was documented as the proximate cause of irreversible graft loss. |
| Renal artery thrombosis | Vascular/thrombotic or microangiopathic |  |
| Renal vein thrombosis | Vascular/thrombotic or microangiopathic |  |
| Thrombosis of graft-supplying vessels, not otherwise specified | Vascular/thrombotic or microangiopathic |  |
| Thrombotic microangiopathy | Vascular/thrombotic or microangiopathic |  |
| Bleeding in the transplant area / graft rupture / procedure-related graft bleeding | Bleeding in transplant bed |  |
| Graft malrotation | Surgical/preservation/gastrointestinal complication |  |
| Cardiac failure or cardiorenal decompensation | Cardiorenal decompensation / cardiac failure | Used only when the cardiorenal course was judged to be the proximate cause of irreversible loss. |
| Preservation injury | Surgical/preservation/gastrointestinal complication |  |
| Transplant hydronephrosis / obstructive urological complication | Urological/hydronephrosis |  |
| Sepsis | Infection/sepsis |  |
| BKV or CMV nephropathy | BKV/CMV nephropathy | Virological detection alone was not equivalent to biopsy-supported viral nephropathy as primary cause. |
| Recurrence of primary renal disease | Recurrent disease |  |
| Bowel perforation or related gastrointestinal surgical complication | Surgical/preservation/gastrointestinal complication |  |
| Unknown or no defensible dominant proximate cause | Unclear/multifactorial | Included unresolved overlapping pathways and primary nonfunction when no identifiable dominant proximate cause could be supported. Primary nonfunction with an identifiable proximate cause was assigned to that cause; its distribution is shown in Table S1D. |

# Table S1C. Mapping of documented causes of DWFG

| **Documented cause of death** | **Cause group used in Table 4** |
| --- | --- |
| Pulmonary embolism | Cardiovascular |
| Myocardial infarction | Cardiovascular |
| Sudden cardiac death | Cardiovascular |
| Mesenteric ischaemia | Cardiovascular |
| Sepsis | Sepsis |
| Intracerebral haemorrhage | Intracerebral haemorrhage |
| Gastrointestinal bleeding | Gastrointestinal bleeding |
| Malignancy | Malignancy |
| Suicide | Suicide |
| Unknown / insufficient documentation | Unknown |

**Table S1D. Assigned primary cause among 21 events with primary nonfunction**

| **Assigned primary cause among events with primary nonfunction** | **n** |
| --- | --- |
| Vascular/thrombotic or microangiopathic | 12 |
| Unclear/multifactorial | 3 |
| Rejection | 2 |
| Cardiorenal decompensation / cardiac failure | 1 |
| Bleeding in transplant bed | 1 |
| Graft malrotation (surgical complication) | 1 |
| Sepsis/infection-associated graft loss | 1 |
| Total | 21 |

Primary nonfunction was recorded as a clinical graft-function phenotype. The table shows the separately assigned proximate primary cause and explains why the 21 primary-nonfunction events do not form an additional mutually exclusive cause category.

**Abbreviations:** BKV, BK polyomavirus; CMV, cytomegalovirus; DCGF, death-censored graft loss; DWFG, death with a functioning graft.
